# Verification: model-free phasing with enhanced predicted models in *ARCIMBOLDO_SHREDDER*

**DOI:** 10.1107/S2059798322009706

**Published:** 2022-10-20

**Authors:** Ana Medina, Elisabet Jiménez, Iracema Caballero, Albert Castellví, Josep Triviño Valls, Martin Alcorlo, Rafael Molina, Juan A. Hermoso, Massimo D. Sammito, Rafael Borges, Isabel Usón

**Affiliations:** a Crystallographic Methods, Institute of Molecular Biology of Barcelona (IBMB-CSIC), Barcelona Science Park, Helix Building, Baldiri Reixac 15, 08028 Barcelona, Spain; b Department of Crystallography and Structural Biology, Institute of Physical Chemistry ‘Rocasolano’, Spanish National Research Council (CSIC), Madrid, Spain; c Department of Biophysics and Pharmacology, Biosciences Institute, São Paulo State University (UNESP), Botucatu, Sao Paulo 18618-689, Brazil; d ICREA, Institució Catalana de Recerca i Estudis Avançats, Passeig Lluís Companys 23, 08003 Barcelona, Spain; Institute of Integrative Biology, University of Liverpool, United Kingdom

**Keywords:** phasing, *ARCIMBOLDO*, *ARCIMBOLDO_SHREDDER*, fragment-based molecular replacement, verification, model bias, predictions, *AlphaFold*, *RoseTTAFold*

## Abstract

Structure predictions have matched the accuracy of experimental structures from close homologues, providing suitable models for molecular replacement phasing. Even in predictions that present large differences due to the relative movement of domains or poorly predicted areas, very accurate regions tend to be present. These are suitable for successful fragment-based phasing as implemented in *ARCIMBOLDO*. The particularities of predicted models are inherently addressed in the new *predicted_model* mode, rendering preliminary treatment superfluous but also harmless. *B*-value conversion from predicted LDDT or error estimates, the removal of unstructured polypeptide, hierarchical decomposition of structural units from domains to local folds and systematically probing the model against the experimental data will ensure the optimal use of the model in phasing. Concomitantly, the exhaustive use of models and stereochemistry in phasing, refinement and validation raises the concern of crystallographic model bias and the need to critically establish the information contributed by the experiment. Therefore, in its *predicted_model* mode *ARCIMBOLDO_SHREDDER* will first determine whether the input model already constitutes a solution or provides a straightforward solution with *Phaser*. If not, extracted fragments will be located. If the landscape of solutions reveals numerous, clearly discriminated and consistent probes or if the input model already constitutes a solution, model-free verification will be activated. Expansions with *SHELXE* will omit the partial solution seeding phases and all traces outside their respective masks will be combined in *ALIXE*, as far as consistent. This procedure completely eliminates the molecular replacement search model in favour of the inferences derived from this model. In the case of fragments, an incorrect starting hypothesis impedes expansion. The *predicted_model* mode has been tested in different scenarios.

## Abstract 2

The accuracy of the models predicted using *AlphaFold* and *RoseTTAFold* eases phasing with molecular replacement and raises the question of model bias. *ARCIMBOLDO_SHREDDER* solves structures while aiming to establish the experimental information in a crystallographic determination by introducing systematic, local verification of phasing solutions.

## 1. Introduction

*AlphaFold* (Jumper *et al.*, 2021 ▸) has brought the accuracy of predictions to a level of atomic detail comparable to that of close homologs, as seen in CASP13 and CASP14 (Kryshtafovych *et al.*, 2019 ▸). When *AlphaFold* or *RoseTTAFold* predictions are successful, solving the crystallographic phase problem by molecular replacement (MR) is facilitated (Baek *et al.*, 2021 ▸; Pereira *et al.*, 2021 ▸; Millán *et al.*, 2021 ▸) provided that the particularities of the models are factored in. *CCP*4 (Winn *et al.*, 2011 ▸) has adapted its programs and developed new tools to make the most of predicted models (McCoy *et al.*, 2022 ▸; Simpkin *et al.*, 2022 ▸; Krissinel *et al.*, 2022 ▸). Even in cases where only part of the predicted model closely represents the structure in the crystal it will be possible to exploit fragments to identify these parts and expand the partial solutions. *ARCIMBOLDO* (Rodríguez *et al.*, 2009 ▸) combines the location of model fragments such as polyalanine helices using *Phaser* (McCoy *et al.*, 2007 ▸) with density modification (Sheldrick, 2002 ▸) and map tracing (Sheldrick, 2010 ▸; Thorn & Sheldrick, 2013 ▸; Usón & Sheldrick, 2018 ▸) using *SHELXE*. Other fragment-based methods, such as *FRAP* (Shrestha & Zhang, 2015 ▸), *AMPLE* (Bibby *et al.*, 2012 ▸) and *MrBUMP* (Keegan & Winn, 2007 ▸), also make suitable use of partial models in combination with density modification.

Modern crystallographic methods have become increasingly integrated and are informed by prior knowledge at all stages (Usón *et al.*, 2021 ▸). The extensive use of practically complete previous models throughout a crystallographic determination (Kovalevskiy *et al.*, 2016 ▸) is combined with a reliance on ideal stereochemistry as the fundamental principle in validation of the experimental outcome (Williams *et al.*, 2018 ▸). This should raise concern, as phases are not experimentally determined in molecular replacement but rather are adopted from the model hypothesis. As a consequence, crystallographic model bias is a known, well documented issue (Bhat, 1988 ▸; Brünger, 1992 ▸; Kleywegt, 2000 ▸). Unequivocally establishing the information contributed by the experiment beyond the model should be a prime objective in a crystallographic determination (Terwilliger *et al.*, 2008 ▸). Fragment-based structure solution was originally introduced as an *ab initio* phasing method, relying on general hypotheses and deriving validation from the fact that correct hypotheses would successfully expand beyond the starting model, providing independent information. *ARCIMBOLDO_SHREDDER* (Sammito *et al.*, 2014 ▸; Millán *et al.*, 2018 ▸) extracts fragments from remote homologs and refines them against the experimental data to identify such successful, starting hypotheses through their expansion. Even with fragments derived from a perfect model, if only their extensions and not the starting hypothesis itself is adopted a model-free phasing is achieved. The present work explores the application of such model-free phasing with predicted models within the workflow of *ARCIMBOLDO_SHREDDER* or directly from a molecular replacement solution. The new implementation can also address multimeric structures. Given a comprehensive, highly accurate model, locating the independent fragments is pointless, but local verification of its features remains informative. Whichever route is followed, after the location of complete or partial models, the traces resulting from the expansion of partial structures from which the starting structure is omitted are combined in our software *ALIXE* (Millán *et al.*, 2020 ▸; Millán, Sammito, Garcia-Ferrer *et al.*, 2015 ▸). This is especially relevant in the typical solution landscape produced by predicted models, characterized by multiple clearly discriminated probes, as opposed to the scarce correct solutions rendered by remote homologs. The present work describes the use of *ARCIMBOLDO_SHREDDER* to solve crystallo­graphic structures with predicted models and introduces model-free verification to probe the local validity of the *ARCIMBOLDO* solution or of external MR solutions. Its use is illustrated and discussed with representative test cases.

## 2. Materials and methods

### 2.1. Computing setup

Structure solutions and tests were run on a local HTCondor v.8.4.5 (Tannenbaum *et al.*, 2001 ▸) grid made up of 160 nodes totalling 225 GFlops and on a 24-core workstation with 128 GB RAM (Intel Xeon CPU E5-2680 v.3) running Linux.

*AlphaFold* predictions were performed on two workstations with the following characteristics: AMD Ryzen Thread Ripper 3975WX, Nvidia GeForce RTX 3090 24 GB. *AlphaFold*2 was run on a virtual machine with an Ubuntu 20.04.4 LTS OS using 48 of the host’s 64 cores and 192 GB of its 256 GB RAM, and also on a workstation with Intel Core i9-9980XE, GeForce GTX 1080 8 GB, 64 GB RAM, Debian 10 (Buster).

### 2.2. External software

*Phaser* (McCoy *et al.*, 2007 ▸) is required to perform the MR search of the fragment models. *Phaser* 2.8.*x* versions from the *CCP*4 (Winn *et al.*, 2011 ▸) and *Phenix* (Liebschner *et al.*, 2019 ▸) distributions were used.

*SHELXE* (Sheldrick, 2010 ▸) is required to provide density modification based on the sphere-of-influence algorithm (Sheldrick, 2002 ▸) and the *SHELXE* 2022 version was used for phase extension and model tracing (Usón & Sheldrick, 2022 ▸). Along with side-chain tracing, this version incorporates masking of the starting-model map region during tracing (parameter -V).

### 2.3. Model prediction

*AlphaFold*2 was used in the cloud through the Google Colaboratory Notebook *MMseqs*2 (Mirdita *et al.*, 2022 ▸), the Google Colaboratory Notebook from DeepMind (Jumper *et al.*, 2021 ▸) or directly from a local installation on a workstation of the code distributed via the repository at https://github.com/deepmind/alphafold (Jumper *et al.*, 2021 ▸). *RoseTTAFold* was used online through the server at https://robetta.bakerlab.org (Baek *et al.*, 2021 ▸).

### 2.4. Figures of merit and phase comparison

The figures of merit used in decision making in the fragment-location and scoring part of the *ARCIMBOLDO* runs described in this work were the *Phaser* intensity-based LLG and *Z*-score (Read & McCoy, 2016 ▸), and the correlation coefficient between observed and calculated normalized intensities (CC; Fujinaga & Read, 1987 ▸) calculated by *SHELXE* (Sheldrick, 2002 ▸).

In order to combine phase sets from partial traces *ARCIMBOLDO* uses *ALIXE* (Millán *et al.*, 2020 ▸). Two indicators are computed to probe consistency: map correlation coefficients (mapCC; Lunin & Lunina, 1996 ▸) and weighted mean phase differences (wMPD). In this study, we will refer to nonrandom solutions whenever their wMPE (mean phase difference to the true phases) is below 80°.

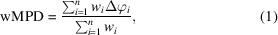




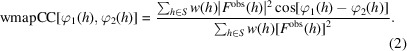




### 2.5. Graphics software

Model and maps were examined with *Coot* version 0.8.7 (Emsley *et al.*, 2010 ▸). Figures were prepared with the *PyMOL* molecular graphics system (version 1.8; Schrödinger) and *Matplotlib* version 1.5.3 (Hunter, 2007 ▸).

### 2.6. Test data and predicted models

**Figure 1:**
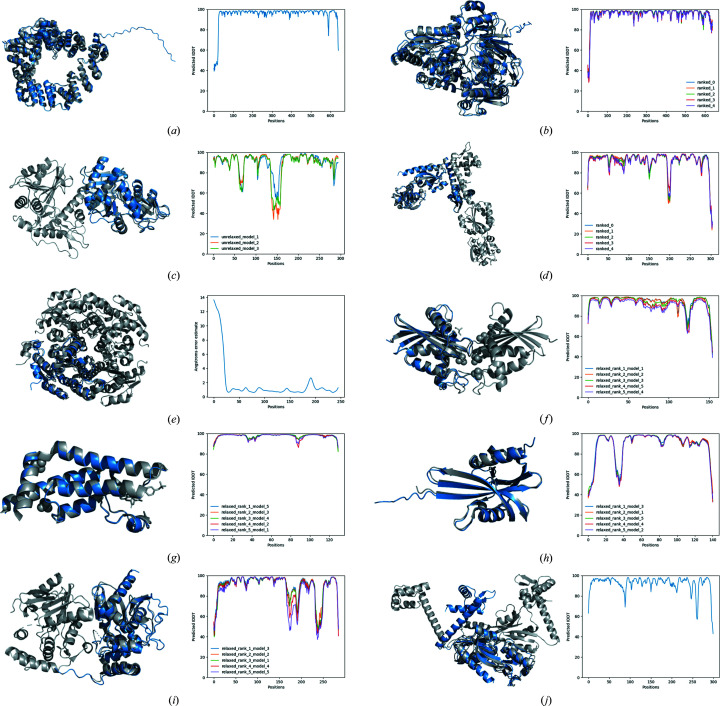
Superposition of the best-ranked model (blue) and the final structure (grey) (left) and quality of the models represented by predicted LDDT or error estimate in Å per position (right). (*a*) PDB entry 5ohu. (*b*) AMIA. (*c*) GLYAT. (*d*) TsaR. (*e*) HheD2. (*f*) PDB entry 7vse. (*g*) PDB entry 7q6t. (*h*) PDB entry 7syc. (*i*) PDB entry 7vo4. (*j*) AtzR.

Representative cases to cover the various uses of model-free verification within *ARCIMBOLDO_SHREDDER*, to validate an already placed MR solution obtained with a predicted model or to produce a solution with a predicted model are described. Structures and models are displayed in Fig. 1 ▸ and their characteristics are summarized in Table 1 ▸.

#### 2.6.1. PDB entry 5ohu

PDB entry 5ohu is a soluble lytic transglycosylase from *Pseudomonas aeruginosa* (Lee *et al.*, 2018 ▸). The structure was originally solved with *ARCIMBOLDO_SHREDDER* (Millán *et al.*, 2018 ▸) in the first implementation of the spherical mode, using the best-scoring model (PDB entry 1qsa; van Asselt *et al.*, 1999 ▸) as identified using *HHpred* (Söding *et al.*, 2005 ▸) with 31% sequence identity. The final solution accomplished a CC of 48% and 563 residues traced in seven chains.

#### 2.6.2. AMIA

PDB entry 8a42 is the crystal structure of a bacterial lipoprotein. The structure was originally solved with *ARCIMBOLDO_SHREDDER* starting from a template with 26% sequence identity. *SEQUENCE SLIDER* (Borges *et al.*, 2020 ▸) was used to extend starting partial polyalanine models with side chains in plausible ways, increasing the signal and revealing the solution after density modification and auto-tracing with *SHELXE*.

#### 2.6.3. GLYAT

GLYAT is a glycine *N*-acyltransferase from *Bos taurus* that catalyses the transmission of an acyl-coA group to a glycine molecule; this reaction participates in the detoxification of xenobiotics, especially benzoic acid. The structure was originally solved by MR with *Phaser* using a model predicted by *RoseTTAFold* (Baek *et al.*, 2021 ▸).

#### 2.6.4. TsaR

PDB entry 3fxq is TsaR, a LysR-type transcriptional regulator (Monferrer *et al.*, 2010 ▸). The structure was originally solved by single isomorphous replacement with anomalous scattering (SIRAS) experimental phasing of a xenon derivative with *SHELXD* (Schneider & Sheldrick, 2002 ▸) and *RESOLVE* (Terwilliger, 2000 ▸).

#### 2.6.5. HheD2

PDB entry 7b73 is a halohydrin dehalogenase (Wessel *et al.*, 2021 ▸). The structure was solved with *ARCIMBOLDO_SHREDDER* by combining fragment-based molecular replacement with density modification.

#### 2.6.6. PDB entry 7vse

PDB entry 7vse is an X-ray structure of *Escherichia coli* ribonuclease HI in complex with Zn^2+^ (Liao *et al.*, 2022 ▸). The structure was solved in 2021 by MR with *Phaser*.

#### 2.6.7. PDB entry 7q6t

PDB entry 7q6t corresponds to the bromo-domain of ATAD2 with AZ13824374 (Winter-Holt *et al.*, 2022 ▸). The structure was solved in 2021 by MR with *AMoRe* (Navaza, 2001 ▸).

#### 2.6.8. PDB entry 7syc

PDB entry 7syc is a nucleoside triphosphate pyrophosphohydrolase from *Klebsiella pneumoniae*. The structure was solved in 2021 by MR with *MoRDa* (Vagin & Lebedev, 2015 ▸).

#### 2.6.9. PDB entry 7vo4

PDB entry 7vo4 is a pimaricin type I PKS thioesterase domain (apo Pim TE; Zhou *et al.*, 2022 ▸). The structure was solved in 2021 by MR with *Phaser*.

#### 2.6.10. AtzR

PDB entry 7z7j is AtzR, a LysR-type transcriptional regulator from *Pseudomonas*. The structure was determined with *ARCIMBOLDO_SHREDDER* using *AlphaFold* models (Castellví *et al.*, 2022 ▸).

### 2.7. Distribution of the software

All of the *ARCIMBOLDO* programs are distributed through the *CCP*4 suite (Winn *et al.*, 2011 ▸) and are available through the PyPI (Python Package Index) project (https://pypi.org/project/arcimboldo/). The software is under the BSD 3-clause licence. Documentation and tutorials can be found on our website (http://chango.ibmb.csic.es/arcimboldo).

## 3. Results and discussion

The *ARCIMBOLDO_SHREDDER* program has been adapted to optimally exploit templates derived from predicted models rather than from experimental structures of homologs, while systematically removing model bias. It is activated by setting the keyword predicted_model to true or selecting the *predicted_model* mode through the interfaces, and operates either on an MR solution obtained with a predicted model or directly on a predicted model. It integrates model-free verification and also incorporates a new general feature to solve structures with multiple copies in the asymmetric unit. A detailed description of the algorithm as well as examples of its application follows.

### 3.1. *Predicted_model* mode implementation

**Figure 2:**
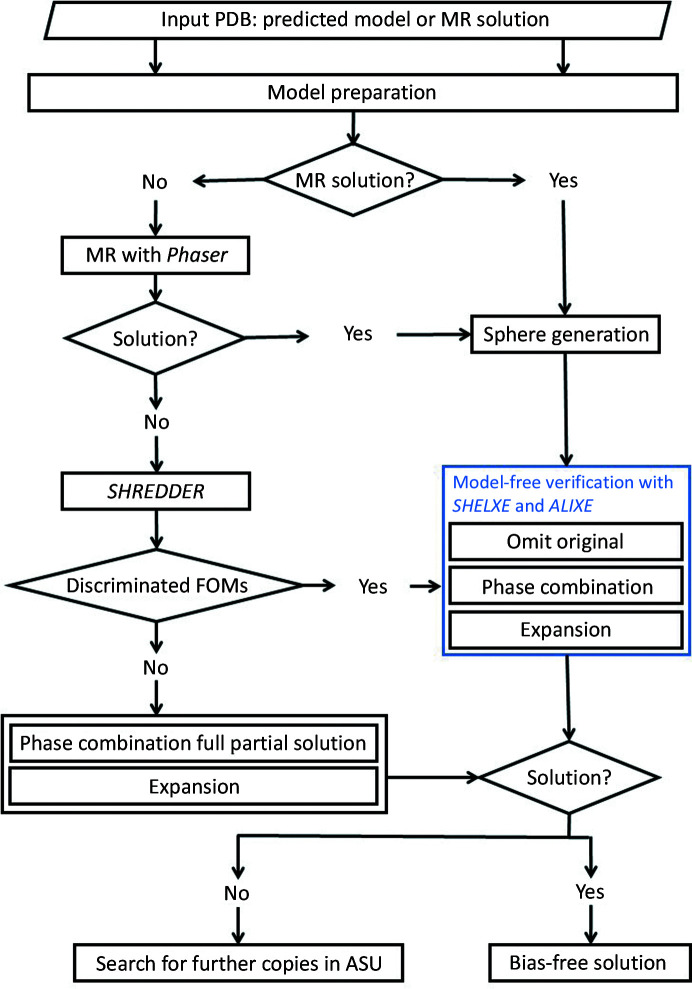
Workflow of *predicted_model* mode. *ARCIMBOLDO_SHREDDER* treats predicted models irrespective of their origin, annotating them with *ALEPH* for further decomposition. It performs a preliminary assessment of the input PDB entry to establish through the CC calculated by *SHELXE* whether it already constitutes a nearly complete solution or whether *Phaser* easily renders one. A solution will be shredded into fragments but unnecessary phasing steps will be skipped up to the start of verification. Otherwise, fragment location and assessment will take place as within the usual *ARCIMBOLDO_SHREDDER* workflow. In multimeric structures, placement of more than one monomer through the multicopy mode could be necessary to reach a complete solution. Solutions are rated by *Phaser* LLG and translation *Z*-score, *SHELXE* CC and consistency. If few or marginal solutions are obtained, their combination in *ALIXE* and expansion with *SHELXE* will proceed as in *ARCIMBOLDO_SHREDDER*. If discrimination is clear or the model provided already constitutes a solution, expansion will be performed enforcing systematic elimination of the starting models to free the structure determination from bias. All fragments will be subjected to expansion, masking the map in the region of the original model and rendering traces exclusively outside this area. All traces will be combined in reciprocal space and the new map will be iteratively modified and traced.

The method is implemented within *ARCIMBOLDO_SHREDDER* spherical mode, which is written in Python 3 and is backwards-compatible with Python 2.7. The current version supports X-ray diffraction data up to 2.5 Å resolution in general cases and to 3.0 Å resolution for coiled coils. Fig. 2 ▸ shows the overall workflow for the *predicted_model* mode, including model-free verification. The procedure starts by assessing whether the input model already constitutes a solution or straightforwardly delivers a solution in *Phaser*, in which case fragment location will be skipped. In either case, model preparation is carried out to eliminate unstructured and disconnected areas and to set *B* values. The model is then annotated for further decomposition with *ALEPH* (Medina *et al.*, 2020 ▸) and decomposed in overlapping spheres of a size guided by the expected LLG in *Phaser* (Oeffner *et al.*, 2018 ▸). One particularity is that annotation will strive to differentiate domains, which will be segregated during sphere generation. Fragments are located if needed, and when the landscape of solutions shows a profusion of clearly discriminated placements their expansion with *SHELXE* through density modification and tracing will omit the original fragments. Only the traces derived from each fragment are retained and combined in reciprocal space with *ALIXE* (Millán *et al.*, 2020 ▸). The resulting clustered phases will be expanded until a full solution is obtained. If the structure is a multimer and expansion of a first placement does not suffice to provide a solution, subsequent copies will be located.

### 3.2. Model preparation: partition and annotation of the template

Molecular replacement is improved when using predicted models though a specific treatment that includes the replacement of the information codified as *B* values [predicted LDDT (Mariani *et al.*, 2013 ▸) or error estimates] with physically sensible *B* values (Baek *et al.*, 2021 ▸; Millán *et al.*, 2021 ▸) and the removal of parts predicted with low confidence above a chosen threshold of pLDDT or error estimates.

The *predicted_model* mode of *ARCIMBOLDO_SHREDDER* entails a particular and automatic preparation of predicted models, which can originate from any available predictor, for example *AlphaFold*, *RoseTTAFold*, *MODELLER* (Webb & Sali, 2021 ▸) or *SWISS-MODEL *(Waterhouse *et al.*, 2018 ▸), or can have been pre-processed for crystallographic use by another program. The predicted models are pre-processed, annotated and decomposed into fragments. Consistent with the rationale in *ARCIMBOLDO* (Millán, Sammito & Usón, 2015 ▸), our method will not rely on pLDDT but rather will let the experimental data select and refine stereochemically sensible input fragments prepared to be comparable. Therefore, it includes a different pre-processing of the *B* factors, and removal of parts predicted with low confidence is performed through *ALEPH*. In most cases, very low pLDDT or high error estimates will correspond to coil regions or unstructured areas, which will be detected and filtered out by *ALEPH*. However, in some cases correct regions might be associated with lower prediction scores, underestimating the quality of the models produced. Instead of using a threshold of pLDDT or error estimate, our approach will take a decision based on the soundness of secondary and tertiary structure and let the measured data probe the local model correctness and refine the model geometry.

The default pre-processing in *ARCIMBOLDO_SHREDDER* trims the side chains to alanine residues and sets a common *B* value of 25 Å^2^ for all atoms, producing a library of fragment models with equivalent scattering. Current versions of deep-learning protein predictions show high side-chain accuracy when the backbone prediction is accurate (Jumper *et al.*, 2021 ▸), so by default side chains from predicted models will be preserved. In these models, H atoms are removed as they are placed at inappropriately long distances for the X-ray scattering experiment and are occasionally named in a way that may lead other programs to interpret them as heavier elements, and the *B* factors are set to a common value of 25 Å^2^ for the main chain and 50 Å^2^ for the side chains.

The secondary- and tertiary-structure elements are identified and annotated with *ALEPH*, relying on the relations among characteristic vectors defined from the centroids of C^α^ atoms to the centroids of carbonyl O atoms from all overlapping tripeptides. In this step, the removal of unstructured areas predicted with low confidence is performed. The strictness thresholds that affect the annotation of secondary-structure elements have been optimized for predicted models.

Decomposition into small, compact folds is predetermined by *ALEPH* through the community-clustering algorithm; this allows the identification of compact rigid groups to refine their relative rotation and translation. For predicted models, a hierarchical decomposition is performed to differentiate domains. Both criteria are combined in the generation of fragments in order to segregate domains.

**Figure 3:**
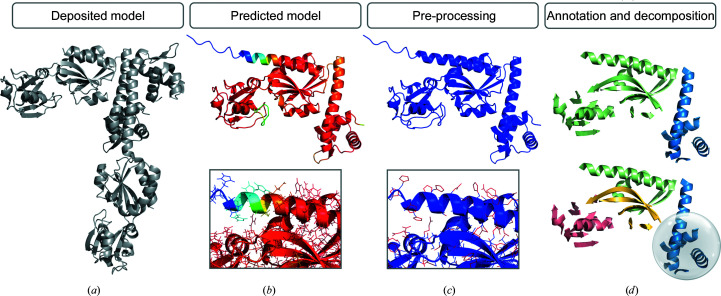
Scheme of model preparation. (*a*) Deposited model as a grey cartoon. (*b*) Predicted model and detail of side chains coloured by pLDDT values (bottom), where red represents high accuracy and blue represents low accuracy. (*c*) Pre-processed model coloured by *B* factors set to a common value of 25 Å^2^ for the main chain and 50 Å^2^ for the side-chain atoms and detail (bottom). (*d*) Annotation and decomposition defined in *ALEPH*: hierarchical decomposition to identify domains restricts sphere generation.

The model preparation is illustrated in Fig. 3 ▸. The performance of the model treatment of the *predicted_model* mode is illustrated through the set of molecular replacement solutions summarized in Table 2 ▸. Structure solution was accomplished with *Phaser*. Comparison of the performance of the predicted models with and without model preparation is shown through the CC and wMPE of the MR solution against the deposited structure. In all cases model processing leads to a CC above 25%, allowing identification of when an MR solution has been input to *ARCIMBOLDO* for validation. In general, the CC increases with model preparation, but when a large amount of correct coil is removed the value may decrease.

### 3.3. Input recognition: MR solution or raw model

*ARCIMBOLDO_SHREDDER* may be employed either to verify a previously placed MR solution obtained with a predicted model or to phase using a predicted model. The program will automatically discern the situation and take the most straightforward route to eliminating model bias if the *predicted_model* mode is selected. When the model provided contains a CRYST1 card coincident with the experimental data it will be tested as a possible solution after model preparation. If the correlation coefficient (CC) between the intensities calculated from the model and the experimental data exceeds 25% (Table 2 ▸), the input will be regarded as a previous solution. Otherwise, the input will be treated as an unplaced model.

### 3.4. Phasing

A preliminary MR search with *Phaser* using the complete model has been incorporated to avoid unnecessary calculations if the predicted model shows high accuracy. If the input already constitutes a solution or the processed model easily renders one, the program will proceed to model-free verification. Otherwise, an *ARCIMBOLDO_SHREDDER* spheres run optimized for predicted models will follow.

In either case, a library of equal-sized spherical fragments of comparable scattering as informed by the eLLG is extracted. Annotation and decomposition of the models in *ALEPH* will be conditioned by domain estimation and the library is reduced to avoid models with disjoint regions or large voids so that the domains are effectively segregated. Fragments will be placed with *Phaser* and the solution will be rated by their LLG after rigid-body refinement, translation *Z*-score, *SHELXE* CC and mutual consistency.

**Figure 4:**
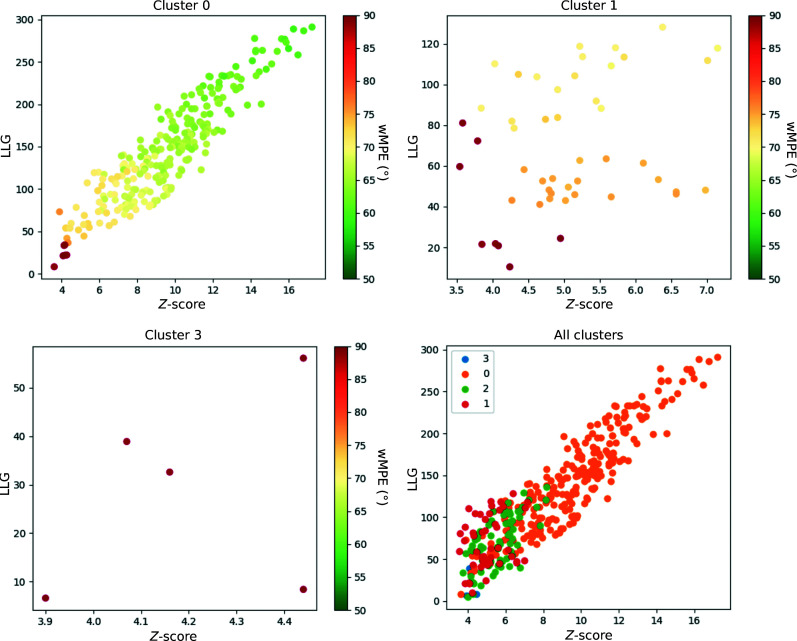
Scatter plots displaying the figure of merit LLG versus translation *Z*-score for partial solutions clustered by the values of the rotation angles in the case of TsaR, representative of the typical landscape occurring when phasing with predicted models. The correctness of the solutions is measured by average phase errors to the deposited structure and is represented in (*a*), (*b*) and (*c*) by the colour scale. The structure contains two copies in the asymmetric unit displaying different conformations, one closer than the other to the search model. (*a*) Rotation cluster containing numerous, well discriminated, correctly placed fragments. (*b*) Rotation cluster with incorrect and marginal solutions. (*c*) Rotation cluster with incorrect solutions. (*d*) Solutions from all clusters (in different colours) clearly set apart the orange cluster shown in (*a*).

Whereas remote homologs typically render few correct partial solutions, which do not stand out amongst incorrect solutions, predicted models tend to reveal numerous, clearly discriminated and consistent probes, as shown for TsaR in Fig. 4 ▸. Given such a landscape of solutions, model-free verification will be activated within the *ARCIMBOLDO_SHREDDER* workflow, enforcing systematic elimination of the starting models to free the structure determination from bias. Otherwise, if few or marginal solutions are obtained, their combination in *ALIXE* and expansion with *SHELXE* will proceed as in the standard *ARCIMBOLDO_SHREDDER* spherical mode.

### 3.5. Model-free verification

Model-free verification may start from many partial solutions produced within *ARCIMBOLDO* or from a distinct full MR solution, in which case the placed model will be fragmented into equal-sized spheres centred on each atom. Depending on the size of the structure more or fewer spheres will be produced and a representative selection will take place covering the full structure. These partial solutions will be subjected to expansion and autotracing in *SHELXE*, masking the map in the region of the original model and rendering traces exclusively outside this area. All consistent traces will be combined in reciprocal space and the new map will be iteratively modified and traced. This procedure completely eliminates the molecular replacement search model in favour of the inferences derived from this model, thus eliminating model bias. In the case of fragments, an incorrect starting hypothesis impedes expansion.

### 3.6. *ARCIMBOLDO* multicopy

If no solution is derived from the placement of a single copy and the asymmetric unit is known to contain a multimer of the search model, a multicopy search will be initiated. To avoid a combinatorial growth of partial solutions, the prioritization involves a search for a second copy of a subset of placed fragments, limited to the rotation and the translation functions followed by the packing filter, which are very fast steps. All probes are then sorted according to their LLG in the translation search. Only the top probes are sent to the time-consuming rigid-body refinement and expansion steps. In cases where the asymmetric unit is known to contain more than two copies of the template, all expected copies for each probe will be placed before the expansion step.

This method is illustrated by the test case of the LysR-type transcriptional regulator AtzR (PDB entry 7z7j), which contains two copies of 300 residues in the asymmetric unit (Castellví *et al.*, 2022 ▸). The *AlphaFold* prediction yielded a model with an overall confidence of 92%. Once solved, superposition revealed an r.m.s.d. between the crystallo­graphic structure and prediction of 4.7 Å for 277 aligned residues.

**Figure 5:**
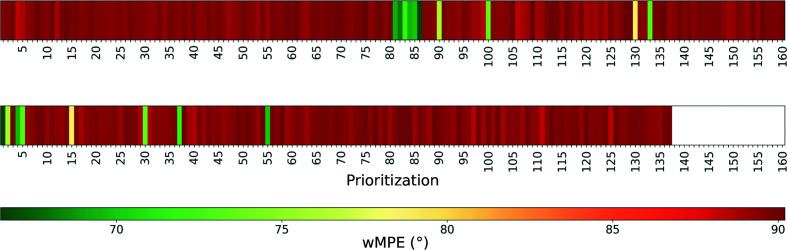
Model prioritization in the multicopy mode of *ARCIMBOLDO*. Plot of the placed partial models ordered after (*a*) placement and rigid-body refinement of the first fragment and (*b*) rotation and translation of the second fragment. The *x* axis represents the priority order characterizing the placed models. The colour scale indicates the correctness of the models according to their wMPE values.

Success in phasing with one copy of this model in *ARCIMBOLDO_SHREDDER* will depend on the parameterization. A retrospective analysis of a run requiring the placement of two copies is shown, quantifying the correctness of the probes against the final refined structure through the wMPE at all stages. In the case of PDB entry 7z7j, after locating the first copy none of the probes was discriminated with prominent figures of merit, although correct placements were present. In particular, ten probes out of 160 were solutions characterized by a wMPE of between 67° and 80°. Their LLG and *Z*-scores did not set them apart from random placements and they would have been ranked in position 81 or below (Fig. 5 ▸ *a*). After the prioritization step based on the translational search for the second fragment, the best model characterized by a wMPE of 67° could clearly be identified according to its figures of merit scoring the top position (LLG = 242 and *Z*-score = 17; Fig. 5 ▸ *b*). Rigid-body refinement and expansion of the top solution revealed the final structure after three cycles of density modification and autotracing. The solution was characterized by a CC of 35% and a wMPE of 45°, with 343 residues traced out of a total of 600.

The multicopy mode has proven to be effective in the solution of a previously unknown structure, a transcriptional repressor from *E. coli* (PDB entry 8a39). Data from crystals belonging to space group *C*2 were collected to a resolution of 2.1 Å (Rojas-Altuve *et al.*, 2011 ▸). The asymmetric unit volume could have hosted four copies of the 327-amino-acid monomer based on cell-content analysis. Thus, the run that solved the structure was parameterized to locate fragments for four copies. After the prioritization step based on the location of the second fragment, location of the third and the fourth copies proceeded followed by expansion with *SHELXE* to build the complete model with side chains. Unexpectedly, the solution revealed that the structure contained only three instead of four monomers in the asymmetric unit.

## 4. Discussion of test cases

Test cases subjected to the procedure described in Fig. 2 ▸ rendered the results summarized in Table 3 ▸. The first five were solved with fragments in *ARCIMBOLDO_SHREDDER*, whereas the remaining cases were solved externally by molecular replacement with *Phaser* and subjected to verification within *ARCIMBOLDO*. These cases were used to develop the workflow and derive default parameterization. Solution after verification is achieved over a broad range of parameterizations, whether through the fragment-phasing route or after fragment decomposition of the MR solution. The accuracy in all or part of the predicted models gives rise to, upon the omit procedure and subsequent phase clustering of the numerous correct solutions, sets characterized by a 20° lower MPE than those clusters of fragments from remote homologs. In the case of PDB entry 5ohu, for example, with fragments from a distant homolog (PDB entry 1qsa) the best wMPE obtained after their combination with *ALIXE* was 64°, whereas combination of the resulting traces after omitting the original fragment from a predicted model rendered a wMPE of 40°. Overall, the final solutions after the verification step are rather complete as further density modification and model building in *SHELXE* improves the solution. Side-chain tracing is activated unless prevented, as the sequence is derived from the model. Other than for model tracing, the results displayed correspond to the defaults that were finally adopted, which as for all *ARCIMBOLDO* programs may be modified by the user. The estimated r.m.s.d. of the search fragments versus the true structure is typically lower than for remote homologs and is accordingly reduced to 0.8 Å. Model decomposition and internal refinement in the *gyre* and *gimble* steps (McCoy *et al.*, 2018 ▸) further decreases it to 0.6 Å. An eLLG target of 60 has been adopted as a default to define the sphere size, unless the size of the template does not support it, in which case it is internally lowered.

## 5. *Predicted_model* mode with coiled coils

Coiled-coil structures pose particular difficulties for phasing that derive from the modulation in their diffraction data (Caballero *et al.*, 2021 ▸), resulting in incorrect partial solutions showing high figures of merit. The *coiled_coil* mode implemented in *ARCIMBOLDO_LITE*, which employs ideal polyalanine α-helices, introduced specific features to overcome this issue, including a verification procedure based on scoring discrimination between apparent solutions and artificially generated solutions (Caballero *et al.*, 2018 ▸). Perturbations mimicking the deviations found in incorrect solutions were induced in the alternatives generated. These attempts to disprove the best solution constituted our first implementation of verification and in the case of coiled coils constitute the optimal way to identify correct solutions, discriminating them from rogue placements. If a predicted coiled-coil structure represents a single helix the specific *coiled_coil* mode in *ARCIMBOLDO_LITE* with model helices should be preferable to the *predicted_model* mode in *ARCIMBOLDO_SHREDDER*. However, a multimeric prediction can be used as a search model in *ARCIMBOLDO_SHREDDER*, retaining its dedicated verification rather than the general model-free procedure. When both *coiled_coil* and *predicted_model* modes are activated, *ARCIMBOLDO_SHREDDER* will follow the model preparation of a predicted model, but the coiled-coil verification of the final solution will be similar to that implemented in *ARCIMBOLDO_LITE*, which will consider the modulation in the data typical of coiled-coil structures.

## 6. Concluding remarks

The high accuracy of the new *AlphaFold* and *RoseTTAFold* predictions has an immediate impact on the way that macromolecular crystallographic determinations can proceed. A successful prediction eases the determination of starting phases provided that the particularities of such models are efficiently addressed. However, using a model at all stages in the interpretation of an experimental determination introduces model bias and the need to separate the knowledge gained from the experiment beyond the model. *ARCIMBOLDO_SHREDDER* addresses both needs in the implementation of its *predicted_model* mode. It processes predicted models irrespective of their genesis or previous pre-processing and segregates domains. If the determination proves challenging on account of the failure of a significant part of the model, achieving extension to a nearly complete structure will verify the starting hypothesis. If the model is close to the final structure, systematic elimination of all fragments used to generate extensions will locally probe every part of the experimental solution. Even though predicted models have the correct sequence and side chains in place, at the end of this verification procedure nothing comes directly from the model, but only from what could be obtained outside model fragments after the expansion step, resulting in ‘model-free’ phasing.

## Figures and Tables

**Table 1:** Test structures and models The r.m.s.d. was calculated with *SUPERPOSE* (Krissinel & Henrick, 2004 ▸). ASU, asymmetric unit.

Structure	Space group	Solvent content (%)	No. of residues	% α	% β	Chains in ASU	Resolution (Å)	Model generation	R.m.s.d. (Å)(No. of residues aligned)	pLDDT or error estimate
PDB entry 5ohu	*P*6_3_	60	642	78	13	1	2.2	*AlphaFold Colab* DeepMind	1.4 (597)	95.1
AMIA (PDB entry 8a42)	*P*2_1_2_1_2_1_	46	638	57	34	1	1.2	*AlphaFold* local workstation	2.2 (603)	96.2
GLYAT	*P*2_1_2_1_2_1_	40	295	50	38	2	1.35	*AlphaFold Colab MMseqs*2	0.7 (290)	90.2
TsaR (PDB entry 3fxq)	*C*2	54	305	57	36	2	1.85	*AlphaFold* local workstation	0.9 (291)	91.9
HheD2 (PDB entry 7b73)	*P*2_1_2_1_2_1_	52	243	57	32	4	1.6	*RoseTTAFold* online server	1.0 (224)	1.8
PDB entry 7vse	*P*222_1_	44	155	45	44	2	2.08	*AlphaFold Colab MMseqs*2	1.0 (149)	94.5
PDB entry 7q6t	*P*6_5_22	70	130	82	13	1	2.05	*AlphaFold Colab MMseqs*2	0.5 (130)	97.8
PDB entry 7syc	*P*6_1_22	61	141	34	57	1	2.0	*AlphaFold Colab MMseqs*2	0.5 (120)	89.5
PDB entry 7vo4	*P*2_1_	48	286	55	35	2	2.1	*AlphaFold Colab MMseqs*2	0.7 (239)	90.3
AtzR (PDB entry 7z7j)	*P*4_1_2_1_2	53	600	56	33	2	1.8	*AlphaFold* local workstation	4.7 (277)	92.0

**Table 2:** Characterization of CC and wMPE for the MR solutions using the predicted models with and without our model preparation

	Without model preparation		With model preparation	
PDB code	CC	wMPE (°)	CC	wMPE (°)
7vse	19.5	52.8	25.6	47.5
7q6t	45.2	34.4	36.2	38.3
7syc	14.7	63.7	35.4	39.5
7vo4	15.7	58.4	27.3	45.9

**Table 3:** Figures of merit for model-free phasing tests Best wMPE fragment refers to the probes before omitting the model. The best wMPE trace was obtained for the traces produced after omitting the original model. wMPE cluster *ALIXE* is for the best phase set combining consistent solutions after model omission. wMPE expansion refers to the final solution. All runtimes correspond to a workstation with 24 cores as described in Section 2.

Structure	Size (amino acids), template/probes	Best wMPE (°), fragment/trace	wMPE cluster *ALIXE* (°)	No. of combined traces	CC expansion (%)	wMPE expansion (°)	Runtime (min)
PDB entry 5ohu	393/91–95	52/53	39	40	45	28	312
AMIA (PDB entry 8a42)	335/88–92	62/49	38	40	48†	19†	723
GLYAT	192/90–94	57/43	33	40	37	25	1020
TsaR (PDB entry 3fxq)	202/84–88	59/52	42	40	44	32	161
HheD2 (PDB entry 7b73)	159/106–110	66/66	48	40	39	24	655
PDB entry 7vse	208/69–73	51/62	53	126	31†	50†	44
PDB entry 7q6t	83/32–36	45/49	31	58	46	28	15
PDB entry 7syc	98/37–41	32/37	29	83	44	27	39
PDB entry 7vo4	337/99–103	52/56	46	66	38	39	84

† Models built with side-chain tracing in *SHELXE*.
